# Beyond the ST segment: Multivessel disease and total coronary occlusion in Non-ST segment elevation myocardial infarction patients in Pakistan; A single center retrospective study

**DOI:** 10.12669/pjms.42.(ICON26).15775

**Published:** 2026-04

**Authors:** Saima Ali, Irsa Nadeem, Aneela Altaf Kidwai, Syed Ghazanfar Saleem

**Affiliations:** 1 Dr. Saima Ali, MBBS, FCPS. Head of Department, Department of Emergency Medicine, Indus Hospital and Health Network, Karachi, Pakistan; 2 Dr. Irsa Nadeem, MBBS. Resident, Department of Emergency Medicine, Indus Hospital and Health Network, Karachi, Pakistan; 3 Dr. Aneela Altaf Kidwai, MBBS, FCPS. Department of Internal Medicine, Abbasi Shaheed Hospital, Karachi, Pakistan.; 4 Dr. Syed Ghazanfar Saleem, MBBS, FCPS. Chair, Department of Emergency Medicine, Indus Hospital and Health Network, Karachi, Pakistan

**Keywords:** Electrocardiography, Multivessel Disease, NSTEMI, Percutaneous coronary intervention, Total coronary occlusion

## Abstract

**Background and Objective:**

Non-ST segment elevation myocardial infarction (NSTEMI) may be associated with multivessel disease (MVD) and total coronary artery occlusion (TO); hallmarks of occlusive myocardial infarction (OMI). The study aimed to determine the frequency and predictors of MVD and TO in NSTEMI patients with OMI at a tertiary care center in Pakistan.

**Methodology:**

This retrospective observational study analyzed the medical records of NSTEMI patients aged > 18 years, who underwent PCI at Indus Hospital and Health Network, Karachi, Pakistan, during 2022. Clinical features, electrocardiograph (ECG) and echocardiographic findings were reviewed. Univariate and multivariate logistic regression identified independent predictors of MVD and TO. Adjusted odds ratio (aORs) and 95% confidence intervals (CIs) were reported.

**Results:**

Among the 671 NSTEMI patients (mean age 56.92 ± 11.29 years, 64% male), MVD and TO was detected in 35.42% and 33.72% respectively. Independent predictors for MVD included age >60 years (aOR 1.44, 95% CI: 1.01-2.04; p=0.045), chest pain at presentation (aOR 1.71, 95% CI: 1.18-2.49; p=0.005) and T-wave inversion (aOR 3.00, 95% CI: 1.92-4.67; p=<0.001). Predictors of TO included age < 60 years (aOR 0.68, 95% CI: 0.48-0.97; p=0.033), ST depression (aOR 1.70, 95% CI: 1.16-2.49; p=0.006) and absence of T-wave inversion (aOR 0.41, 95% CI: 0.27-0.62; p<0.001).

**Conclusion:**

A significant subset of NSETMI patients in Pakistan present with underlying OMI. Simple clinical and ECG features may be helpful in identification of such high-risk cases in resource-limited settings; enabling timely intervention and improved outcome.

## INTRODUCTION

Cardiovascular disease (CVD) remains the leading cause of global mortality, accounting for nearly 18 million deaths annually.^1^ The burden of early and aggressive CVD is particularly high in South Asia and literature shows that Pakistani adults develop CVD at least a decade earlier than their Western counterparts. 2 Diabetes mellitus (DM), hypertension (HTN), and dyslipidemia contribute to extensive atherosclerotic involvement in our population. Hence, CVD is one of the primary contributors of mortality and disability-adjusted-life-years (DALY) in Pakistan.^3^

In CVD, acute coronary syndromes (ACS) encompass ST-elevation myocardial infarction (STEMI), non-ST elevation myocardial infarction (NTEMI), and unstable angina. Conventionally, STEMI was considered pathognomonic of total coronary occlusion (TO), warranting immediate reperfusion therapy. However, through improved diagnostic sensitivity and review of changing risk profiles in recent years, multivessel disease (MVD) and TO are also observed in patients with NTEMI.^4^ Recent data suggest that a substantial subset of NSTEMI patients have a completely occluded culprit artery; a condition called occlusive myocardial infarction (OMI).^5^,^6^ These patients are often under-recognized since ST-elevation, the primary trigger for urgent percutaneous coronary intervention (PCI) is missing. Subsequent delays in revascularization result in larger infarct size, higher rates of heart failure, and increased mortality.^7^

Compounded by limited access to diagnostic and interventional cardiology services, timely triage and diagnosis in the emergency department (ED); high-risk NSTEMI patients, often times are not managed according to best practices and clinical guidelines in Pakistan.^8^ Within this context, there is an urgent need to refine risk stratification tools that can help distinguish patients with NSTEMI who are likely to have OMI. Literature highlights clinical and electrocardiographic indicators, such as age, typical chest pain, ST-segment depression, and T-wave inversion, that may help predict underlying coronary artery occlusion.^9^,^10^ However, such findings have not been well-validated in the Pakistani setting.

The study aimed to determine the frequency and predictors of MVD and TO in NSTEMI patients with OMI who presented at the Indus Hospital and Health Network (IHHN) ED, Karachi, Pakistan in 2022.

## METHODOLOGY

This retrospective study was conducted at IHHN in 2022 after obtaining institutional board approval (IHHN_IRB_2022_12_005). Medical records of all adult patients > 18 years who presented with NSTEMI at the ED and underwent PCI were reviewed. Patients with STEMI and new-onset left bundle branch block were excluded.

Data were extracted from the electronic health record (EHR). Indirect variables included patient demographics, clinical history (e.g., diabetes mellitus, hypertension, smoking), vital signs, and history of cardiac disease- myocardial infarction (MI), PCI or coronary artery bypass grafting (CABG). NSTEMI was diagnosed based upon high-sensitivity troponin-I (11-34 ng/L),^11^ along with one or more of the following; ischemic symptoms (e.g., chest pain), ST depression or T-wave inversion on ECG, or new regional wall motion abnormalities and low ejection fraction (EF) 12 on echocardiography. The outcome variable was the presence of MVD or TO identified during PCI.

Data were analyzed using STATA version 16. Continuous variables were reported as means ± standard deviation (SD) or medians (interquartile range, IQR) based on normality (assessed using the Shapiro-Wilk test). Categorical variables were reported as frequencies and percentages. Binary logistic regression was performed to identify predictors of MVD and TO.

Univariate logistic regression was first performed to assess the association between potential predictors and each outcome. Variables with p < 0.25 in univariate analysis, along with clinically relevant covariates (e.g., age, gender, comorbidities) were included into a multivariate logistic regression model using backward stepwise selection method.^13^ This approach allowed systematic elimination of non-significant variables while retaining those with independent associations.

To address confounding, known clinical risk factors were prioritized in model inclusion regardless of univariate significance. The final models reported adjusted odds ratio (aOR) with 95% confidence interval (CI), and p < 0.05 was considered as statistically significant.

### Patient consent:

Participant consent was not sought in view of the retrospective chart review through EHR. Data were only accessible to the principal investigator (PI) and the research team. All efforts were made to adhere to strict global standards of medical research while analyzing and reporting the results.

## RESULTS

**Fig.1:**
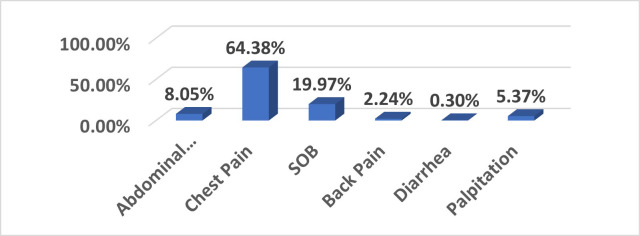
Distribution of ED presenting complaints

A total of 671 NSTEMI patients met the inclusion criteria. The mean age was 56.92 ± 11.29 years; and 64% (n=429) were male. Most patients presented with chest pain (64.4%), followed by shortness of breath (20.0%) and abdominal pain (8.1%) (Fig.1).

Hypertension and DM were highly prevalent, affecting 67% and 76% of the cohort respectively. Ischemic heart disease (IHD) was noted in 39.8%, while 10.3% had history of prior myocardial infarction (MI). ST depression (68.0%) and T-wave inversion (20.5%) were the most common ECG abnormalities. On echocardiography, 96.9% had regional wall motion abnormality and 85.7% had reduced EF (Table-I).

**Table-I:** Descriptive statistics of the study population (n=671).

Variable		n (%)
Gender	Male	429 (64)
	Female	242 (36)
Comorbidities	Diabetes mellitus	507 (75.56)
	Hypertension	449 (66.92)
	Ischemic heart disease	267 (39.79)
	Chronic kidney disease	17 (2.53)
	Asthma	4 (0.60)
	Cerebrovascular accident, Hypothyroidism, Hyperthyroidism, Tuberculosis, Deep venous thrombosis	1 (0.15)
Electrocardiographic findings	ST depression	456 (67.96)
	T wave inversion	138 (20.50)
	Biphasic T waves	42 (6.26)
	QT prolongation	19 (2.83)
	Q waves	8 (1.19)
	Left bundle branch block, Poor R wave progression	5 (0.75)
	Right bundle branch block, sinus bradycardia	2 (0.30)
	Left/ right axis deviation, Wellen’s sign	1 (0.15)
Cardiac activity on echocardiography	Hypokinetic	574 (85.67)
	Akinetic	72 (10.75)
	Normal	24 (3.58)

**Fig.2:**
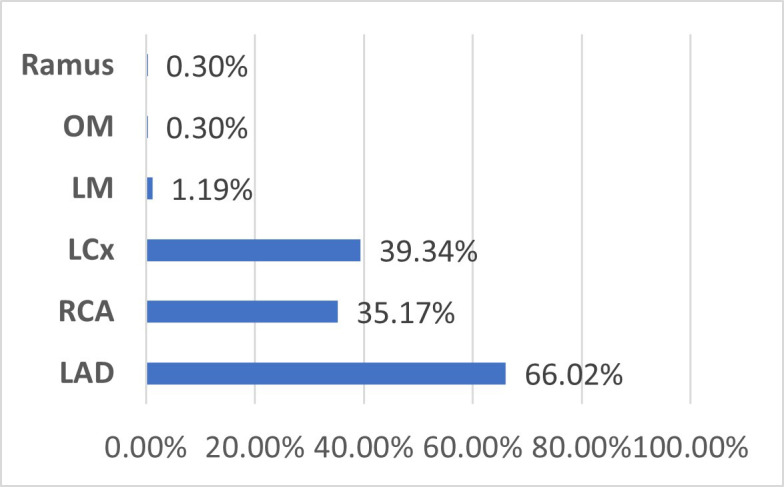
Pattern of coronary artery occlusion *LAD: Left anterior descending coronary artery, **RCA:** Right coronary artery, **LCX:** Left circumflex artery, **OM:** Obtuse marginal artery, **LA:** Left coronary artery

Multivessel disease, defined as significant stenosis in two or more major coronary vessels, was observed in 35.4% of the patients. Total coronary occlusion (TO) was identified in 33.7%. The left anterior descending (LAD) artery was the most occluded culprit vessel, involved in in 66.0% of TO cases, followed by left circumflex (LCX) (39.0%) and right coronary artery (RCA) (35.0%) (Fig.2).

In multivariate logistic regression, three factors were independently associated with MVD- age >60 years (aOR 1.44, 95% CI: 1.01-2.04; p=0.045), chest pain at presentation (aOR 1.71, 95% CI: 1.18-2.49; p=0.005) and T-wave inversion (aOR 3.00, 95% CI: 1.92-4.67; p <0.001). QT prolongation was negatively associated with MVD (aOR 0.09, 95% CI: 0.02-0.43; p=0.002). Hypertension showed a borderline association (p=0.080) (Table-II).

**Table-II:** Predictors of multivessel disease (MVD) in NSTEMI patients

Predictor variables	Adjusted Odds ratio (aOR)	95% Confidence interval	p- value
Age > 60 years	1.44	1.01–2.04	0.045
Chest pain at presentation	1.71	1.18–2.49	0.005
T-wave inversion	3.00	1.92–4.67	<0.001
QT prolongation	0.09	0.02–0.43	0.002
Hypertension	1.39	0.96–2.03	0.080
Diabetes mellitus	0.84	0.79–1.72	0.434
Male gender	1.11	0.77–1.61	0.568

Predictors of TO included younger age < 60 years (aOR 0.68, 95% CI: 0.48-0.97; p=0.033), ST depression (aOR 1.70, 95% CI: 1.16-2.49; p=0.006), and absence of T-wave inversion (aOR 0.41, 95% CI: 0.27-0.62; p<0.001). Biphasic T-waves (aOR 0.44; p=0.041), chest pain (aOR 0.66; p=0.019), and hypertension (aOR 0.61; p=0.006) were inversely associated with TO. Diabetes showed a positive trend but was not statistically significant (Table-III).

**Table-III:** Predictors of total coronary artery occlusion (TO) in NSTEMI patients

Predictor variables	Adjusted Odds ratio (aOR)	95% Confidence interval	p- value
Age > 60 years	0.68	0.48–0.97	0.033
ST depression	1.70	1.16–2.49	0.006
T wave inversion	0.41	0.27–0.62	<0.001
Biphasic T waves	0.44	0.20–0.97	0.041
Chest pain at presentation	0.66	0.46–0.93	0.019
Hypertension	0.61	0.43–0.87	0.006
Diabetes mellitus	1.44	0.96–2.17	0.080

## DISCUSSION

The study highlights the significant burden of high-risk angiographic findings among NSTEMI patients in Pakistan. Over one-third of the study population had MVD or TO leading to OMI, despite the absence of ST-elevation on ECG. These findings mirror global data, suggesting that OMI in NSTEMI is not uncommon and should be considered in decision-making.^14^-^16^

Older age and classical symptoms were associated with MVD, consistent with chronic atherosclerotic burden.^17^ T-wave inversion significantly increased the odds of MVD. Conversely, the absence of T-wave inversion along with ST-depression were significant predictors of OMI, likely reflecting ischemia in posterior or diffuse territories.^18^ This highlights the importance of timely ECG interpretation in managing NSTEMI patients in the ED.

Younger patients and those without HTN had higher odds of TO, possibly due to acute plaque rupture and limited collateral circulation, as previously reported.^19^ Atypical symptoms and absence of HTN were also identified as risk factors for OMI, suggesting that clinical vigilance is needed, even when patients do not present with typical features of MI.

In our study, the LAD artery was the most frequently occluded vessel in NSTEMI patients, seen in 62% of cases with TO. This finding is consistent with regional data from South Asia, where aggressive anterior coronary disease is common.^20^ Factors such as earlier onset atherosclerosis and clustering of metabolic risk contribute to LAD predominance. Globally, however, the culprit artery in NSTEMI varies. Studies from the Western population often report higher left circumflex (LCX) artery or RCA involvement. These differences may stem from genetic, lifestyle, and diagnostic disparities.^21^ Recognizing this variation is essential for tailoring stratification and intervention strategies across populations.

The findings from this study are particularly relevant in Pakistan, where late presentations and limited PCI availability may delay intervention in high-risk NSTEMI patients. In resource-constrained setups, relying on nuanced ECG interpretation and clinical clues becomes essential. Training ED physicians and nurses to recognize ECG findings suggestive of OMI can guide urgent transfers and reduce time to reperfusion.^22^

### Limitations

This single-center retrospective study may not be generalizable to rural populations or patients managed without angiography. Confounding variables such as the timing of symptom onset, socioeconomic factors, and medication adherence were not recorded. Long-term outcomes post-discharge were also not evaluated. However, this is among the first studies till date to report angiographic predictors or OMI in NSTEMI in Pakistan and sets the stage for prospective multicenter research.

## CONCLUSION

A substantial proportion of NSTEMI patients in this cohort had either MVD or TO, findings traditionally associated with STEMI. ECG features such as T-wave inversion and ST segment depression, along with age and symptomatology, can help identify these high-risk cases. Early recognition and consideration for urgent PCI, even in the absence of ST-elevation, may improve outcomes in this vulnerable population.

### Recommendations

Future research should include multicenter, prospective designs to enhance generalizability and capture regional variations across Pakistan. Incorporating patients managed conservatively or presenting late would offer a more comprehensive view of NSTEMI presentations. Long-term follow-up is needed to assess the prognostic impact of MVD and TO. Developing bedside risk scores using clinical and ECG predictors could aid early identification of OMI. Training frontline physicians to recognize high-risk NSTEMI patterns is critical in resource-limited settings. Finally, improving public awareness and strengthening emergency cardiac networks may be the key to reducing delays in care.

### Authors’ Contribution:

**SA:** Study concept, design, and critical manuscript review.

**IN:** Data collection, analysis, literature search, and manuscript writing.

**AAK:** Data interpretation and manuscript editing.

**SGS:** Supervision, study design feedback, and final approval of the version to be published.

All authors have read the final version and are responsible and accountable for the accuracy and integrity of the work.
